# The Information Loss Problem and Hawking Radiation as Tunneling

**DOI:** 10.3390/e27020167

**Published:** 2025-02-05

**Authors:** Baocheng Zhang, Christian Corda, Qingyu Cai

**Affiliations:** 1 School of Mathematics and Physics, China University of Geosciences, Wuhan 430074, China; 2 Department of Mathematics and Physics, SUNY Polytechnic Institute, Utica, NY 13502, USA; cordac.galilei@gmail.com; 3 Center for Theoretical Physics, Hainan University, Haikou 570228, China; qycai@hainanu.edu.cn; 4 School of Information and Communication Engineering, Hainan University, Haikou 570228, China; 5 Peng Huanwu Center for Fundamental Theory, Hefei 230026, China

**Keywords:** entropy, correlation, information loss, Hawking radiation

## Abstract

In this paper, we review some methods that have tried to solve the information loss problem. In particular, we revisit the solution based on Hawking radiation as tunneling and provide a detailed statistical interpretation of the black hole entropy in terms of the quantum tunneling probability of Hawking radiation from the black hole. In addition, we show that black hole evaporation is governed by a time-dependent Schrödinger equation that sends pure states into pure states rather than into mixed states (Hawking had originally established that the final result would be mixed states). This is further confirmation of the fact that black hole evaporation is unitary.

## 1. Introduction

Due to Stephen Hawking’s work, it became known that when general relativity is combined with quantum theory, black holes can emit thermal radiation, which is known as Hawking radiation [1,2]. Shortly afterward, Hawking discovered that regardless of the initial state that forms the black hole, it will evolve into a thermal radiation state or a mixed state. This many-to-one evolution not only violates the principle of unitarity in quantum mechanics but also leads to the loss of information about the black hole’s initial state, which is known as the famous “black hole information loss paradox” [3]. Since the discovery of the “black hole information loss paradox”, many scientists have studied the issue, but so far, no solution has completely resolved the problem. This is partly due to the lack of a complete theory of quantum gravity, and partly because even within the framework of semi-classical gravity, the existing solutions still face insurmountable or unresolved issues. Past solutions can generally be categorized into the following several approaches.

The first perspective argues that information is fundamentally lost. The main proponent of this view was Hawking, but in 2005, he published an article stating that information is not lost (“elementary quantum gravity interactions do not lose information or quantum coherence”) [4]. However, since no specific analysis or calculation process was provided, this claim was not widely accepted. Recently, Unruh and Wald [5] reanalyzed and summarized this viewpoint, arguing that the evolution from a pure state to a mixed state during the black hole collapse and evaporation process would not have any impact on existing physics, so information loss is acceptable. However, this analysis of the consequences remains speculative and does not fundamentally demonstrate that information is indeed lost. Whether a true theory of quantum gravity still suggests information loss remains unclear.

The second perspective suggests that information is still conserved in semi-classical theory. Semi-classical theory refers to using classical theory to describe gravity while using quantum theory for everything else. The most straightforward view in this regard is that the problem of information loss should not exist at all, as Peres and Terno stated at the end of their review (“There is no issue of information loss at all”) [6]. However, their analysis of the events seen by different observers is not detailed enough to resolve all the potential paradoxes revealed by the information loss problem.

An important recent development in the international study of the black hole information loss problem is the work of scientists like Almheiri, Engelhardt, and Penington [7,8], who used the AdS/CFT approach to find a way to calculate the entropy of Hawking radiation in a class of special black holes, proving that the entropy of Hawking radiation during the evaporation process follows the Page curve. This is an elegant solution, and this approach has recently attracted considerable attention, as noted in related discussions [9,10]. However, upon closer analysis, it becomes clear that this approach is still essentially semi-classical in nature. Although this solution is mathematically impressive, the physical shortcomings are also evident. The primary issue is that it relies on analyzing quantum extremal surfaces in higher-dimensional spacetime, and whether this extra dimension actually exists remains unclear. Setting aside the calculation method itself, it is still unclear how exactly information is encoded within the black hole and how it emerges from the black hole. Of course, this is a common flaw in all attempts to resolve the black hole information loss problem within the framework of semi-classical theory.

The third perspective considers possible effects beyond current understanding to solve the black hole information loss problem. In this regard, some scientists suggest that black holes do not completely evaporate and may leave behind remnants or transform into a “baby universe” [11] to preserve the relevant information. This idea has some merit and remains an area of ongoing research, as noted in recent review articles [12].

Other scientists propose that black holes may possess not only mass, charge, and angular momentum but also additional degrees of freedom on their surface known as “quantum hair”, which could store information [13]. However, if this method is used to solve the black hole information loss paradox, a mechanism would be needed to transition quantum gravity theory to low-energy local quantum field theory, and this mechanism is not yet fully understood. Notably, the recent study by Hawking and others that black holes possess “soft hair” could potentially aid progress on this issue [14].

Another intriguing approach involves quantum information theory, specifically related to quantum teleportation. However, the method known as “final state projection” [15], which is associated with this concept, does not explain why or how a projection occurs at the final stage of black hole evolution. A more radical idea is the so-called “firewall hypothesis” [16], which maintains the principle of unitarity in quantum mechanics by violating the equivalence principle. Nevertheless, the firewall itself presents a dilemma: either it truly exists, or an alternative solution (see the “ER=EPR” conjecture [17]) must be found. For further solutions and related discussions, see recent reviews [18,19,20].

All those methods mentioned above are based on the thermal spectrum discovered by Hawking, but recently, Parikh and Wilczek [21] developed a method of Hawking radiation as tunneling with the energy conservation considered, which broke down the fixed spacetime background and included the reaction in their method, and thus the result of black hole radiation was obtained as non-thermal naturally. Building on the non-thermal black hole radiation spectrum, it has been demonstrated that this spectrum not only contains correlations that can carry information but also that the total entropy (i.e., the sum of the entropy of the remaining black hole and the radiation) during the black hole radiation process remains conserved [22]. These results are entirely consistent with the requirements of quantum mechanical unitarity, suggesting that the non-thermal radiation process based on energy conservation considerations may indeed be unitary [23]. The fundamental physical picture in this solution is that for black holes with the same mass but different initial states, their radiation processes differ. The number of different radiation processes can reveal the number of microscopic states contained within the black hole. We used standard statistical methods to demonstrate that this number of microscopic states can explain the area entropy of the black hole.

Whether it is the entanglement entropy of the radiation process satisfying the Page curve or the total entropy conservation of the black hole and radiation, both only demonstrate that the radiation process is unitary. It seems challenging to go further. This suggests that new elements need to be introduced in future analyses. As previously discussed, the issue of unitarity also involves a microscopic understanding of black hole entropy. In this paper, we will briefly review how Hawking radiation, viewed as a tunneling process, can demonstrate that the entire radiation process is unitary and provide a microscopic explanation of black hole entropy based on the probabilistic nature of the radiation process itself.

## 2. Hawking Radiation as Tunneling

Here, we will review the mechanism to relate the reaction in the black hole evaporation with particle annihilation. The reaction considered firstly in the calculation of black hole radiation is derived from the tunneling method of Parikh and Wilczek [21]. In their method, they transformed the Schwarzschild coordinates

(1) $$ds^{2}=-\left(1-\frac{2M}{r}\right)dt^{2}+{\left(1-\frac{2M}{r}\right)}^{-1}dr^{2}+r^{2}d\Omega^{2}$$

into the Painlevé coordinates

(2) $$ds^{2}=-\left(1-\frac{2M}{r}\right)dt^{2}+2\sqrt{\frac{2M}{r}}dtdr+dr^{2}+r^{2}d\Omega^{2},$$

which is non-singular at the horizon r=2M. Then, one could consider that the tunneling of particles as emission impetus and calculate the probability as

(3) $$P∼\exp\left[-8\pi E\left(M-\frac{E}{2}\right)\right]=\exp\left(\Delta S\right)$$

where *E* is the energy of tunneling particle and $S=4\pi M^{2}$ is the black hole’s Bekenstein–Hawking entropy. From Equation (3), it is clear that black hole radiation is a non-thermal spectrum, which differs from the thermal spectrum originally obtained by Hawking. The main reason for this difference is that, in Hawking’s calculation of black hole radiation, the reaction effect or the energy conservation was not taken into account. The spacetime background he used was fixed, and thus his calculation may not have been entirely rigorous. The result of thermal radiation he derived might be corrected by considering the reaction effect. In fact, this is indeed the case. When the reaction effect is considered, Parikh and Wilczek recalculated the black hole radiation and found that the final radiation spectrum is not purely thermal. Their calculation is based on the quantum mechanical tunneling effect, where vacuum field fluctuations outside the black hole event horizon generate particle–antiparticle pairs. The antiparticles, or negative energy particles, will tunnel into the black hole, while the positive energy particles escape the black hole, forming black hole radiation. The tunneling barrier is caused by the reduction in the black hole’s mass or by the tunneling particles themselves. When the black hole mass is large, this non-thermal spectrum can be approximated as a thermal spectrum and yields the same temperature as Hawking’s result. However, when the black hole mass is not very large, the non-thermal nature of the spectrum becomes evident.

Since this spectrum is non-thermal, it is natural for us to ask whether there is some correlation hidden in this radiation spectrum. This leads to the question of how to determine whether a correlation exists. In statistics, we determine if two events are correlated by checking whether the probability of each event occurring individually is equal to the probability of both events occurring simultaneously. If they are equal, then the two events are statistically independent; otherwise, there must be a correlation between them. We can understand this correlation in a simpler ways. For example, if there are *m* black balls and *n* white balls in a box, and if we draw a black ball first and then put it back, and then draw a white ball, it is clear that these two events are completely independent. However, if we do not put the black ball back after the first draw, then there is some correlation between the two events. In the context of black holes, after the first particle is radiated, it will certainly not fly back into the black hole. Therefore, the second particle that is radiated will definitely have some correlation with the first particle. Standard statistical analysis shows that such a correlation exists in the non-thermal spectrum given in Equation (3). Why, then, is there no such correlation in the thermal spectrum? This is because, when deriving the thermal spectrum, the recoil effect is not considered. This is equivalent to assuming that after the first particle is radiated, it flies back into the black hole before the second particle is radiated. Clearly, in this case, there would be no correlation between the two radiation events.

Now, we consider a subsequent emission. At first, we will investigate whether there are correlations existing between the two emissions with energies $E_{1}$ and $E_{2}$, respectively. When the first emission with the energy $E_{1}$ finishes, the tunneling probability for a particle of energy $E_{2}$ has to be treated carefully, since the correlation can be assumed in advance. According to the statistic theory described in the last paragraph, the two probabilities can be obtained by taking the integral of their joint probability $P\left(E_{1},E_{2}\right)$, i.e., $P\left(E_{1}\right)=\int P\left(E_{1},E_{2}\right)dE_{2}$ and $P\left(E_{2}\right)=\int P\left(E_{1},E_{2}\right)dE_{1}$, where $P\left(E_{1},E_{2}\right)=P\left(E_{1}+E_{2}\right)$ is the joint probability of the two emissions with energies $E_{1}$ and $E_{2}$ occuring simultaneously. Thus, we can confirm that the correlation exists in the non-thermal radiation spectrum by finding the conditional probability $P\left(E_{2}|E_{1}\right)=\frac{P\left(E_{1},E_{2}\right)}{P\left(E_{1}\right)}\neq P\left(E_{2}\right)$, or by $P\left(E_{1},E_{2}\right)\neq P\left(E_{1}\right)\cdot P\left(E_{2}\right)$. From the perspective of the statistics, the correlation can be measured as follows [24]:

(4) $$C\left(E_{1}+E_{2};E_{1},E_{2}\right)=\ln P\left(E_{1},E_{2}\right)-\ln\left[P\left(E_{1}\right)\cdot P\left(E_{2}\right)\right].$$

Since the correlation exists, we will continue to ask: can this correlation carry information? Can it carry all the information? Our answer is yes. This question involves the concept of mutual information in quantum information theory. Mutual information describes the amount of information that can be shared between two correlated events. Thus, it requires one to calculate that the correlation *C* is equal to the mutual information. For a general composite quantum system composed of subsystems *A* and *B*, the mutual information is defined as S(A:B)≡S(A)+S(B)−S(A,B)=S(A)−S(A|B), where S(A|B) is the conditional entropy [25]. For the situation of black hole tunneling radiation, the entropy for the tunneling emitted particle is obtained as $S\left(E_{i}|E_{1},E_{2},\ldots ,E_{i-1}\right)=-\ln P\left(E_{i}|E_{1},E_{2},\ldots E_{i-1}\right)$, where $E_{i}$ is the energy of the tunneling particle after the black hole has emitted particles with a total energy $E_{f}=\sum_{j=1}^{i-1}E_{j}$. Obviously, this is a conditional entropy that measures the entropy of emission $E_{i}$, given that the values of all the emitted particles with energies $E_{1},E_{2}$, *…* and $E_{i-1}$ are known. Through the calculation, it is not hard to find that the entropy for the remaining black hole with mass $M-E_{f}$ decreases compared with the initial entropy of the black hole, because the emitted particles carry entropies. This balances the total entropy of the black hole and the radiation, and will not lead to any violation of the thermodynamic second law for a black hole [26]. When mutual information is applied to the emissions of two particles with energies $E_{1}$ and $E_{2}$, we have

(5) $$S\left(E_{2}:E_{1}\right)\equiv S\left(E_{2}\right)-S\left(E_{2}|E_{1}\right)=-\ln P\left(E_{2}\right)+\ln P\left(E_{2}|E_{1}\right).$$

Then, it is found that $S\left(E_{2}:E_{1}\right)=8\pi E_{1}E_{2}$, which answers the questions raised at the beginning of this paragraph. Alternatively, we can understand this from another perspective: the lack of information manifests as an increase in uncertainty about an event. In other words, because we do not know the exact mechanical process when a coin is tossed, we cannot accurately predict which side will land face up. However, we all believe that the information about which side will land up is certainly embedded in some complex process. For the black hole radiation process, the correlation we have discovered will ultimately counteract the increased uncertainty due to the lack of information, leading to the possibility that the entire process is unitary.

The discovery of the correlation provides us with a channel through which we can understand the information leakage of black holes. However, there is another question: does entropy remain non-increasing? In information theory, entropy is a measure of uncertainty, but this measure cannot be directly linked to correlation. Therefore, we must examine whether the entropy of the black hole is conserved throughout the entire process, which is the essential requirement for the unitarity of the quantum mechanics. According to the above analysis, except for the first radiation, the other radiations occur in the form of conditional probabilities. Therefore, the entropy carried away by them is also conditional entropy. In this way, we can calculate the total entropy of black hole radiation as

(6) $$S\left(E_{1},E_{2},\cdots ,E_{n}\right)=\sum_{i=1}^{n}S\left(E_{i}|E_{1},E_{2},\cdots ,E_{i-1}\right),$$

where $M=\sum_{i=1}^{n}E_{i}$ corresponds to the energy of all emissions due to the energy conservation. Calculate the summation in Equation (6), and we obtain $S\left(E_{1},E_{2},...,E_{n}\right)=4\pi M^{2}$, which is just the black hole’s Bekenstein–Hawking entropy. Our calculation shows that the continuous tunneling black hole radiation process is an entropy-conserving process. Thus, we can say that the black hole radiation process is a unitary process, and information is not lost.

In particular, our analysis is still phenomenological and does not involve any microscopic mechanisms. However, our analysis is meaningful, as it applies to different types of black hole radiation. On one hand, it indicates that energy and momentum conservation is key to solving the information loss problem. More importantly, it also suggests that regardless of the microscopic mechanism, the spectrum ultimately obtained should be this non-thermal spectrum; otherwise, it could lead to the violation of information conservation.

## 3. Statistic Interpretation of Entropy

Since the radiation process is unitary, it is significant to understand the black hole entropy based on Hawking radiation as tunneling. This can be understood by counting the number of ways the black hole emits radiations [22,27,28]. The so-called ways refer to the following: the black hole first radiates a particle with energy $E_{1}$, then it radiates a particle with energy $E_{2}$, followed by a particle with energy $E_{3}$, and so on, until the black hole has radiated all its energy. Actually, the energy of the particle radiated in the first event could be $E_{1}^{\prime }\neq E_{1}$, the energy of the particle radiated in the second event could be $E_{2}^{\prime }\neq E_{2}$, and so on, until the black hole has radiated all its energy. This is a different radiation way compared to the former. Note that as long as there is one difference in the energy of emitted particles, it can be defined as a new radiation way.

We denote each radiation way as a microstate $\left(E_{1},E_{2},\cdots ,E_{n}\right)$ and $\sum_{i}E_{i}=M$. It is not hard to obtain the probability for the microstate $\left(E_{1},E_{2},\cdots ,E_{n}\right)$ as $P_{t}=P\left(E_{1}\right)*P\left(E_{2}\right)*\cdots *P\left(E_{n}\right)$ with $P\left(E_{1}\right)=\exp\left(-8\pi E_{1}\left(M-E_{1}/2\right)\right)$, $P\left(E_{2}\right)=\exp\left(-8\pi E_{2}\left(M-E_{1}-E_{2}/2\right)\right)$, ⋯, $P\left(E_{n}\right)=\exp\left(-8\pi E_{n}\left(M-E_{1}-E_{2}-\cdots -E_{n-1},E_{n}/2\right)\right)=\exp\left(-4\pi E_{n}^{2}\right)$. After a detailed calculation, we can obtain

(7) $$P_{t}=\exp\left(-4\pi M^{2}\right)=\exp\left(-S_{BH}\right),$$

where $S_{BH}$ is the entropy of the black hole. Further, we can obtain the number of the microstates as $\Omega =\frac{1}{P_{t}}=\exp\left(S_{BH}\right)$, according to the fundamental postulate of statistical mechanics that all microstates of an isolated system are equally likely. This provides a feasible interpretation for the Bekenstein–Hawking entropy $S_{BH}$, that is $S=\ln\Omega =S_{BH}$, in terms of the number of ways for evaporation according to Boltzmann’s definition. On one hand, this provides a statistically microscopic explanation for the entropy of a black hole; on the other hand, it also shows that black hole radiation can carry away all the entropy of the black hole, satisfying the requirement of unitarity in quantum mechanics.

## 4. Time-Dependent Schrödinger Equation for Black Hole Evaporation

The fact that successive emissions of Hawking particles are countable, as has been shown in the previous sections, is consistent with what was originally found by Bekenstein in 1974 [29], that the energy spectrum of a black hole is discrete. Bekenstein indeed obtained $E_{n}=\sqrt{\frac{n}{2}}$ by using the Bohr–Sommerfeld quantization condition because he argued that the Schwarzschild black hole behaves as an adiabatic invariant. A similar result was found in [30], starting from the quantization of the famous Oppenheimer–Snyder gravitational collapse:

(8) $$E_{n}=-\sqrt{\frac{n}{4}}.$$

In quantum mechanics, time evolution of perturbations can be described by an operator [30]

(9) $$U\left(t\right)=\begin{array}{c} W(t)\;\;\;for\;0\leq t\leq \tau \\ 0\;\;\;for\;t<0\;and\;t>\tau . \end{array}$$

Then, the complete (time-dependent) Hamiltonian is described by the operator [30]

(10) H(r,t)≡V(r)+U(t),

where V(r) is given by Equation (48) in [30]. Thus, considering a wave function ψ(r,t), we can write the correspondent *time-dependent Schrödinger equation* for the system (i.e., the evaporating black hole) as [30]

(11) $$i\frac{d|\psi (r,t)>}{dt}=\left[V(r)+U(t)\right]\left|\psi \left(r,t\right)>=H\left(r,t\right)\right|\psi \left(r,t\right)>.$$

The state which satisfies Equation (11) is [30]

(12) $$|\psi \left(r,t\right)>=\sum_{n}a_{n}\left(t\right)\exp\left(-iE_{n}t\right)|\varphi_{n}\left(r\right)>,$$

where the $\varphi_{n}\left(r\right)$ are the eigenfunctions of the time-independent Schrödinger equation in Equation (49) in [30], and $E_{n}$ are the corresponding eigenvalues. Now, we closely follow [30]. In the basis $|\varphi_{n}\left(r\right)>$, the matrix elements of W(t) can be written as

(13) $$W_{ij}\left(t\right)\equiv A_{ij}\delta \left(t\right),$$

where $W_{ij}\left(t\right)=<\varphi_{i}\left(r\right)\left|W\left(t\right)\right|\varphi_{j}\left(r\right)>$ and the $A_{ij}$ are real. In order to solve the complete quantum mechanical problem described by the operator (10), we need to find the probability amplitudes $a_{n}\left(t\right)$ due to the application of the perturbation described by the time-dependent operator (9), which represents the perturbation associated to the emission of a Hawking particle. For t<0 (i.e., before the perturbation operator (9) starts to work), the system is in a stationary state $|\varphi_{m}\left(t,r\right)>,$ at the quantum level m, with energy $E_{m}=-\frac{1}{2}\sqrt{m},$ given by Equation (8). Thus, in Equation (12), only the term

(14) $$|\psi_{m}\left(r,t\right)>=\exp\left(-iE_{m}t\right)|\varphi_{m}\left(r\right)>,$$

is not null for t<0. This implies $a_{n}\left(t\right)=\delta_{nm}\;\;$ for t<0. When the perturbation operator (9) stops working (i.e., after the emission), for t>τ, the probability amplitudes $a_{n}\left(t\right)$ return to being time independent, having the value $a_{m\to n}\left(\tau \right)$. In other words, for t>τ, the system is described by the *wave function $\psi_{final}\left(r,t\right)$*, which corresponds to the state

(15) $$|\psi_{final}\left(r,t\right)>=\sum_{n=1}^{m}a_{m\to n}\left(\tau \right)\exp\left(-iE_{n}t\right)|\varphi_{n}\left(x\right)>.$$

Therefore, the probability to find the system in an eigenstate having energy $E_{n}=-\sqrt{n}$, with n<m for emissions, is given by

(16) $$\Gamma_{m\to n}\left(\tau \right)={\left|a_{m\to n}\left(\tau \right)\right|}^{2}.$$

By using a standard analysis, we obtain the following differential equation from Equation (15):

(17) $$i\frac{d}{dt}a_{m\to n}\left(t\right)=\sum_{l=1}^{n}W_{ml}a_{m\to l}\left(t\right)\exp\left[i\left(\Delta E_{l\to n}\right)t\right].$$

To first order in U(t), by using the Dyson series [30], the solution is obtained as

(18) $$a_{m\to n}=-i\int_{0}^{t}\left\{W_{nm}\left(t^{\prime }\right)\exp\left[i\left(\Delta E_{m\to n}\right)t^{\prime }\right]\right\}dt^{\prime }.$$

Now, we insert Equation (13) in Equation (18), obtaining

(19) $$a_{m\to n}=iA_{nm}\int_{0}^{t}\left\{\delta \left(t^{\prime }\right)\exp\left[i\left(\Delta E_{m\to n}\right)t^{\prime }\right]\right\}dt^{\prime }=\frac{i}{2}A_{nm}.$$

This equation can be combined with Equation (144) in [30] and with Equation (16). We obtain

(20) $$\begin{array}{c} \alpha \exp\left[16\pi \left(n-m\right)\right]=\frac{1}{4}A_{nm}^{2}\\ A_{nm}=2\sqrt{\alpha }\exp\left[8\pi \left(n-m\right)\right]\\ a_{m\to n}=-i\sqrt{\alpha }\exp\left[8\pi \left(n-m\right)\right]. \end{array}$$

As $\sqrt{\alpha }∼1,$ we find $A_{nm}∼10^{-11}$ for n=m−1, (i.e., when the probability of emission has its maximum value). This implies that second-order terms in U(t) are $∼10^{-22}$ and that we can, in turn, neglect them. Clearly, for n<m−1, we get a better approximation, because the $A_{mn}$ are even smaller than $10^{-11}$. Hence, we can write down the final form of the ket representing the state as

(21) $$|\psi_{final}\left(r,t\right)>=\sum_{n=1}^{m}-i\sqrt{\alpha }\exp\left[8\pi \left(n-m\right)-iE_{n}t\right]|\varphi_{n}\left(r\right)>.$$

The state (21) represents a *pure final state instead of a mixed final state.* Therefore, the states are obtained in terms of a *unitary* evolution matrix instead of a density matrix, and this confirms the fundamental conclusion argued in previous sections that information is not lost in black hole evaporation. This result is consistent with ’t Hooft’s idea that Schrödinger equations can be used universally for all dynamics in the universe [30] and dismisses the claim of Hawking that the final result of black hole evaporation would be mixed states [3]. The final state of Equation (21) is due to potential arbitrary transitions m→n, with m>n. Then, the subsequent *collapse of the wave function* to a new stationary state, at the quantum level *n*

(22) $$|\psi_{n}\left(r,t\right)>=\exp\left(-iE_{n}t\right)|\varphi_{n}\left(r\right)>,$$

implies that the wave function of the infalling particle in Hawking’s mechanism of particle creation by black holes has been transferred to the black hole excited state at the quantum level *n* [30], and it is given by

(23) $$|\psi_{\left(m\to n\right)}\left(r,t\right)>\equiv \exp\left(-iE_{n}t\right)\left|\varphi_{n}\left(r\right)>-\exp\left(-iE_{m}t\right)\right|\varphi_{m}\left(r\right)>.$$

This wave function result is entangled with the wave function of the particle, which propagates towards infinity. Clearly, the evolution of black hole evaporation that has been discussed in this section is *unitary*.

## 5. Discussion and Conclusions

It is noted that the discussion about the information loss problem based on our method above has been extended to many different types of black holes [31,32,33,34,35,36,37,38,39,40,41,42] and many different situations [43,44,45,46,47,48,49,50,51,52,53,54,55,56,57,58,59,60,61,62] (also see the review paper [63]). All of these show that the Hawking radiation as tunneling should be a unitary process.

However, when we consider that information can be carried out by Hawking radiation, there is still another question that needs to be resolved: Where is the information stored before it is carried out by Hawking radiation? Before Hawking discovered black hole radiation, the renowned American general relativity expert Wheeler provided an explanation for classical black holes swallowing information: the information is stored inside the black hole—although it is not lost, it is impossible for an external observer to retrieve it. In this view, when the black hole does not radiate or has not yet completed its radiation, people assume that the information remains stored within the black hole. However, can the information actually be inside the black hole? This question is extremely difficult to answer. First, it is not clear what exactly exists inside a black hole, but some studies on black hole volume offer intriguing insights. In 2015, Christodoulou and Rovelli showed that a black hole formed through collapse has an extremely large volume [64]. Whether there are enough degrees of freedom in such a large volume to store information had been studied. It indicated that the degrees of freedom within the black hole’s volume can indeed statistically produce entropy proportional to the black hole’s surface area, but this entropy is much smaller than the black hole’s actual entropy [65,66,67]. Therefore, the black hole’s interior likely does not have enough degrees of freedom to store all the information. However, this does not completely rule out the possibility that information is stored inside the black hole [68].

As for the possibility that information is stored on the event horizon, this is considered more likely, given that black hole entropy is proportional to its surface area. Discussions involving area quantization and entanglement entropy also support this possibility. In this context, the idea of “quantum hair” [13] is worth mentioning, particularly the recent conclusion by Hawking and others that black holes possess “soft hair” [14]. Based on the idea of “soft particles” in quantum field theory and the BMS symmetry of asymptotically flat spacetime, they found that “soft hair” exists in black hole spacetime and can carry information. When this “soft hair” on the black hole’s event horizon is excited, it could explain the black hole’s area entropy. However, whether this “soft hair” carries information about the black hole’s initial state or whether this information can be retrieved by external observers remains unclear.

Another question is whether Hawking radiation truly has a non-thermal spectrum, as suggested by Parikh and Wilczek. This may need to be answered through observation or experimentation. In the context of astrophysics, Hawking radiation is almost unobservable due to its extremely low temperature (for example, the radiation temperature of a black hole with the mass of the Sun is approximately $10^{-7}$ K, which is seven orders of magnitude lower than the 2.7 K temperature of the cosmic microwave background).

A recent interesting approach should also be noted, originally carried out by Vaz in 2014 [69] and recently further developed by one of us (CC) [70,71]. In fact, in 2014, Hawking [72] proposed that BH event horizons could not be the final result of the gravitational collapse. He speculated that the BH event horizon should be replaced by an “apparent horizon” where infalling matter is suspended and then released. Hawking did not give a mechanism for how this can work, which was later given by Vaz [69], who supported Hawking’s conclusion. Vaz indeed discussed an interesting quantum gravitational model of inhomogeneous dust collapse by showing that continued collapse to a singularity can only be achieved by combining two independent and entire solutions of the Wheeler–DeWitt equation [69]. He argued that such a combination is forbidden, leading in a natural way to matter condensing on the apparent horizon during quantum collapse, forming a thin and dense spherically symmetric shell [69]. A similar result had already been obtained by Einstein in 1939 [73]. In [70,71], it was shown that these thin and dense spherically symmetric shells have quantum properties and obey the Schrodinger equation in the non-relativistic case and the Klein–Gordon equation in the relativistic case. Well-defined energy spectra correspond to these equations. Furthermore, the equations themselves were derived from the historical homogeneous Oppenheimer–Snyder gravitational collapse. Nontrivial consequences emerge from these results: (i) black holes have neither horizons nor singularities; (ii) there is neither information loss in black hole evaporation, nor black hole complementarity, nor firewall paradox. Thus, in this case, the information problem is, in principle, solved by showing that BHs actually have a different physical structure, that is, the structure of normal bodies emitting radiation from their surface like any other body. Something similar also happens in the so-called “fuzzball paradigm” [74]. Fuzzballs are objects predicted by string theory, intended to provide a full BH quantum description. Recalling that Bekenstein entropy is obtained through the count of brane bound states, the fuzzball construction of BH microstates implies that these states have no horizon and radiate from their surface like a normal body, so there is no information paradox [74]. In this framework, quantum gravity effects modify the entire region inside the horizon. Thus, one finds a fuzzball, that is, a ball of stringy matter with no horizon. Fuzzballs radiate from their surface like normal bodies, that is, not by the creation of pairs from the vacuum. Thus, one finds no information paradox. The fuzzball paradigm arises from the discovery of D-branes and the BH construction in terms of bound states of branes. An estimate of the radius of brane bound states gives a result of the order of the horizon radius. Several families of brane bound states can be, in turn, constructed, by finding a “fuzzball” (i.e., an object with no horizon, see [74] for details). What the Einstein–Vaz shells and fuzzballs have in common is that quantum gravity corrections become necessary at the horizon scale rather than at the Planck scale, as is believed by most researchers. In other words, quantum corrections to classical general relativity depend on an energy scale rather than a distance scale.

Over the past 40 years, scientists have discovered that it is possible to simulate analogue gravity [75] in many physical systems. In recent years, significant progress has been made on the experimental front, especially with Bose–Einstein condensates, where Hawking radiation has reportedly been observed [76]. Our previous analysis provided an experimental signal [77] for Hawking radiation as a tunneling process. We hope that future experiments can observe the exact form of Hawking radiation in more detail, which would be of great significance in solving the black hole information loss problem.

## Data Availability

No new data were created or analyzed in this study. Data sharing is not applicable to this article.
